# Programmable Shunt Valves for Pediatric Hydrocephalus: 22-Year Experience from a Singapore Children’s Hospital

**DOI:** 10.3390/brainsci11111548

**Published:** 2021-11-22

**Authors:** Min Li Tey, Lee Ping Ng, David C. Y. Low, Wan Tew Seow, Sharon Y. Y. Low

**Affiliations:** 1 Neurosurgical Service, KK Women’s and Children’s Hospital, 100 Bukit Timah Road, Singapore 229899, Singapore; minli.tey@mohh.com.sg (M.L.T.); ng.lee.ping@kkh.com.sg (L.P.N.); 2 Department of Neurosurgery, National Neuroscience Institute, 11 Jalan Tan Tock Seng, Singapore 308433, Singapore; david.low.c.y@singhealth.com.sg (D.C.Y.L.); seow.wan.tew@singhealth.com.sg (W.T.S.); 3 SingHealth Duke-NUS Neuroscience Academic Clinical Program, 11 Jalan Tan Tock Seng, Singapore 308433, Singapore

**Keywords:** pediatric hydrocephalus, programmable shunt valve, ventriculoperitoneal shunt

## Abstract

(1) Background: pediatric hydrocephalus is a challenging condition. Programmable shunt valves (PSV) have been increasingly used. This study is undertaken to firstly, to objectively evaluate the efficacy of PSV as a treatment modality for pediatric hydrocephalus; and next, review its associated patient outcomes at our institution. Secondary objectives include the assessment of our indications for PSV, and corroboration of our results with published literature. (2) Methods: this is an ethics-approved, retrospective study. Variables of interest include age, gender, hydrocephalus etiology, shunt failure rates and incidence of adjustments made per PSV. Data including shunt failure, implant survival, and utility comparisons between PSV types are subjected to statistical analyses. (3) Results: in this case, 51 patients with PSV are identified for this study, with 32 index and 19 revision shunts. There are 3 cases of shunt failure (6%). The mean number of adjustments per PSV is 1.82 times and the mean number of adjustments made per PSV is significantly lower for MEDTRONIC™ Strata PSVs compared with others (*p* = 0.031). Next, PSV patients that are adjusted more frequently include cases of shunt revisions, PSVs inserted due to CSF over-drainage and tumor-related hydrocephalus. (4) Conclusion: we describe our institutional experience of PSV use in pediatric hydrocephalus and its advantages in a subset of patients whose opening pressures are uncertain and evolving.

## 1. Introduction

Hydrocephalus is the most prevalent neurosurgical problem encountered in the pediatric population [1,2,3]. Affected children represent a disproportionate share of all admissions to hospital [4]. The most frequent method of cerebrospinal fluid (CSF) diversion is the ventriculoperitoneal shunt (VPS) [2]. Although the VPS extends survival and leads to improved neurological outcomes, it has notable shortcomings that may compromise patients’ quality of life [2]. Modifications have been developed in the existing shunt systems, in order to circumvent problems associated this treatment modality [5]. For example, programmable shunt valves (PSV) have been increasingly used for patients with hydrocephalus. This is because incorporating a valve with an adjustable opening pressure has the advantage of enabling neurosurgeons to make non-invasive alterations in the opening pressure of the valve as the patient’s clinical course temporally changes after VPS insertion [6,7,8]. Nonetheless, clinical studies on the efficacy of PSV have been conflicting—some have reported no significant differences in shunt failure rates between PSV versus traditional fixed pressure valves (FPV) in children [2,9], while others have demonstrated otherwise [5,10]. Presently, there exists no definite consensus regarding which type of shunt is preferred in the pediatric population, and under what circumstances they should be considered [9,11,12,13,14,15,16,17,18,19].

As part of the largest children’s hospital in Singapore, our Neurosurgical Service is no stranger to the challenges of managing children with shunts. This is particularly relevant for patients who undergo physiological changes that may affect CSF drainage rates during their lifetimes. The primary aims of this study are to review firstly, the use of PSV as a treatment modality for pediatric hydrocephalus; and next, the associated patient outcomes at our institution. Secondary aims include the evaluation of reasons for our use of PSV and corroboration of our results with published literature.

## 2. Materials and Methods

### 2.1. Study Design and Patient Demographics

This is an ethics-approved, retrospective study of prospectively collected data conducted in KK Women’s and Children’s Hospital (SingHealth Centralised Institutional Review Board, CIRB Reference: 2020/2416). The inclusion criteria encompass all patients less than 18 years old who are diagnosed with hydrocephalus and subsequently had a PSV inserted as part of their VPS construct. In addition, patients who failed other previous techniques of CSF diversion and underwent insertion of VPS with a PSV are included. Patients above the age of 18 years, those who do not have hydrocephalus, and patients with incomplete clinical information are excluded. Demographic data is retrieved from the hospital’s electronic medical records and, or hardcopy notes. For the purposes of this study, we define ‘index shunts’ as VPS inserted for the first time in individual patients, including those who failed prior other techniques of CSF diversion, including endoscopic third ventriculostomy (ETV). Next, ‘revision shunts’ refer to VPS inserted after their previous implant(s) have failed, regardless of the underlying cause.

### 2.2. Incidence of Opening Pressure Adjustments in PSV

**Figure 1:**
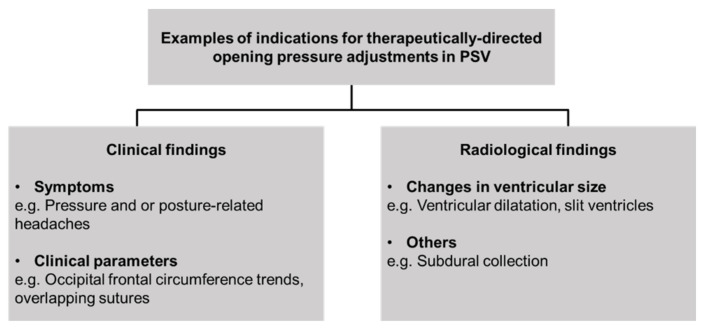
General overview of common indications for adjustments to PSV in study population.

As part of the study, the cumulative number of opening pressure adjustments made per PSV after shunt insertion is tabulated. Here, only adjustments that are therapeutically directed (i.e., based on patient’s clinical and, or radiological indications) are included. (Figure 1). Adjustments made in cases of prior inadvertent opening pressure changes (such as, after exposure during MRI scans) are excluded. The endpoint of this subgroup analysis is to determine whether the use of PSV in each patient has been appropriate. In the context of this study, this translates to the assumption that a higher number of shunt adjustments means that this subset of patients has appropriately benefitted from the use of PSV, in comparison to being initially fitted with FPV.

### 2.3. Statistical Methods

Statistical analyses are generated using SPSS version 27 (Statistical Package for the Social Sciences Statistics, IBM, USA). The Pearson chi-square test or Fisher exact test is used to compare categorical variables, as appropriate. The Wilcoxon-signed ranked test is used to compare continuous variables. Odds ratios (ORs) and 95% confidence intervals (CIs) for risk factors of VPS failure and malfunction are calculated by univariate logistic regression analyses. The Kaplan-Meier method is used to estimate the overall time to shunt failure, and the log-rank test is used to compare the different brands of PSV and failure difference between index and revision-type PSV shunts. A *p*-value of <0.05 is considered statistically significant for this study.

## 3. Results

### 3.1. Overview of Study Population and its Demographics

A total of 396 patients are identified for this study from 1 January 1997 to 31 May 2021. In this cohort, there are 51 cases of PSV insertion (12.8%). This compares with 345 cases of FPV shunts, and 88 cases of endoscopic third ventriculostomy (ETV). Next, the youngest patient with a PSV is 2 months old, while the oldest is 18 years old (mean age = 6 years 6 months old; median age = 4 years old). More PSV are inserted in males (29 cases; 59% of all PSV patients) than in females (20 cases; 41% of all PSV patients). 

**Figure 2:**
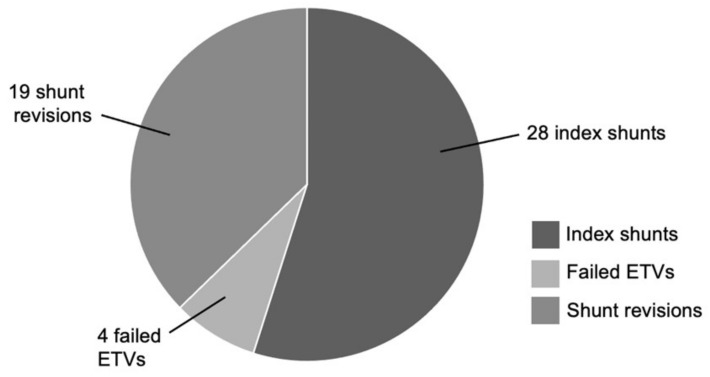
Breakdown of programmable shunt cases.

**Figure 3:**
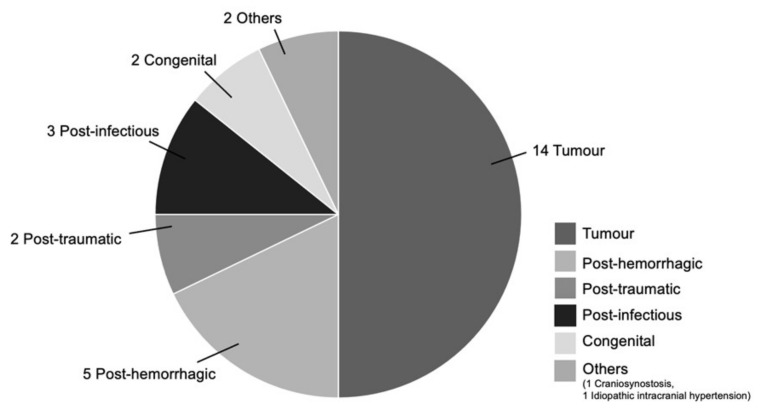
Indications for index PSV insertion.

In the PSV group (*n* = 51), 32 of these cases are index shunts (55%), whereby 4 patients (8%) have prior failed ETVs. The remaining 19 cases are shunt revisions (37%), that is, for either a previous FPV or PSV shunt. (Figure 2). For the index PSV insertion group, the most common indication for shunt placement is that of tumor-related causes (14 cases; 50% of index PSV patients), followed by 5 cases of post-hemorrhagic hydrocephalus (18% of index PSV patients). Here, the primary causes of hemorrhage include: 3 neonatal hemorrhage (2 intraventricular and 1 intraparenchymal hemorrhage), 1 cerebral arteriovenous malformation-related hemorrhage and 1 midbrain cavernoma hemorrhage. There are 3 cases of post-infectious hydrocephalus, 2 post-traumatic hydrocephalus, 2 cases of congenital hydrocephalus, and 1 case of idiopathic intracranial hypertension and craniosynostosis each. (Figure 3). At the time of this writing, there are 18 patients who are no longer on active follow-up with us: 5 patients have been transferred under the care of other institutions (2 patients to our adult hospital, and 3 patients who returned overseas). In addition, 7 patients passed away due to conditions unrelated to shunt malfunction, while no data is available for the 6 remaining patients. The mean duration of follow-up for all PSV shunt patients is 39.7 months (range 1 month to 144 months).

**Figure 4:**
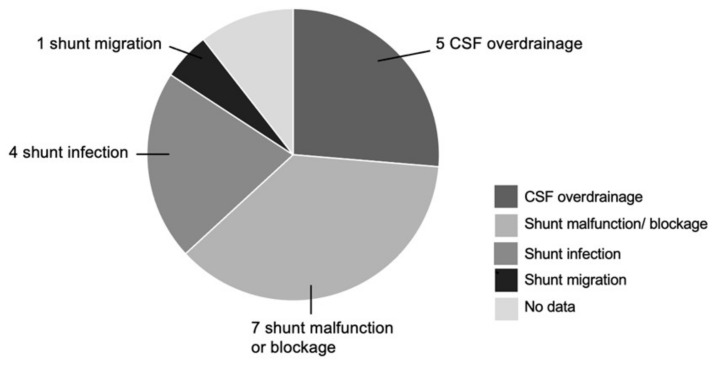
Breakdown of 19 cases with previous shunts.

Among the 19 cases of shunt revision, 2 patients have previous PSV that are revised again to new PSV shunts, while the remaining 17 initially have FPV that are converted to PSV. The indications for shunt revision include 5 cases of CSF over-drainage, 7 cases of shunt malfunction and or, blockage, 4 cases of shunt infection, and 1 case of shunt migration. There are 2 cases where no clinical data is available. (Figure 4). Among the 4 cases of failed ETV, 3 of these cases are performed for tumor-related hydrocephalus (1 medulloblastoma, 1 pineoblastoma, 1 patient with Neurofibromatosis Type 1 (NF1) who presents with concurrent supra- and infratentorial glial tumors) while 1 is attempted for post-hemorrhagic hydrocephalus. Of note, the child with NF1 has ETV attempted twice prior to PSV insertion. The other 3 cases have PSV insertion after failure of their first ETV.

### 3.2. Types of PSV Encountered and Shunt Failure Rate

The most common PSV utilized in our unit are the CODMAN™ Certas (23 cases) and the MEDTRONIC™ Strata (15 cases), followed by the MIETHKE™ ProGAV (9 cases). There are 4 patients (3 CODMAN™ Hakim and 1 Sophysa Polaris^®^) whose shunts are inserted elsewhere but transferred to us for continuity of care. Of note, our neurosurgical team is cognizant of these other types of PSV, especially with regards to their working mechanics, adjustment tools and contactable vendor expertise to ensure patient safety in times of emergency (Table 1).

**Figure 5:**
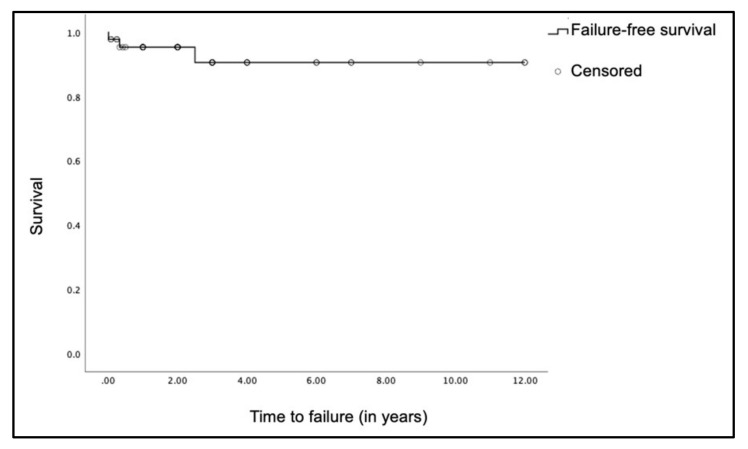
Failure-free survival for all PSV shunts.

Amongst our 51 cases of PSV patients, there are 3 cases of shunt failure (6%) related either to shunt infection or malfunction (Figure 5). These patients subsequently underwent shunt revision, either to a FPV (1 case) or another PSV (2 cases). Here, 1 patient has a history of post-hemorrhagic hydrocephalus with interval shunt infection approximately 2 and a half years later; and decision is made to revise the shunt with a PSV. Another patient with obstructive hydrocephalus secondary to craniosynostosis developed PSV shunt blockage, 8 months after insertion. This shunt is subsequently changed to another PSV while awaiting definitive surgery for the craniosynostosis. The last patient initially has a PSV shunt, but this is changed 4 days after insertion to a FPV shunt due to shunt malfunction. Following that, we compared the time to shunt failure between the different types of PSVs, and time to shunt failure between index and revision for all our PSV shunts. However, there was no statistical significance for both comparisons. (Supplementary Figures S1 and S2).

### 3.3. Analysis of Frequency of PSV Adjustments

In the cohort of 33 PSV patients with available follow-up data, the mean number of adjustments per PSV is 1.82 times (SD ± 1.74). (Supplementary Figure S3). In this case, 22 patients (66.7%) have their opening pressure settings adjusted more than once, whereas the remaining 7 patients (21.2%) required no adjustments of their PSV. Separately, we note that the number of adjustments made per PSV increased in recent years, particularly from 2016 onwards. There is a significantly lower number of mean number of adjustments made per PSV from the period of 1 January 2000–31 December 2015 versus 1 January 2016–30 June 2021 (0.8 mean adjustments per PSV, SD ± 0.9 versus 2.1 adjustments per shunt, SD ± 1.7; *p* = 0.031) (Figure 5). Next, we found that the mean number of adjustments made per PSV is significantly lower for the MEDTRONIC™ Strata PSVs compared with other PSV types (0.8 vs 2.1; *p* = 0.031). We postulate that this may be related to a lower range of settings available for the MEDTRONIC™ Strata, which allows a lesser ‘fine-tuning’ effect, in comparison to the other PSV types. (Table 2). Nonetheless, we acknowledge that the MEDTRONIC™ Strata is the only PSV type available at our institution prior to 2016; with the other 2 brands (CODMAN™ Certas and MIETHKE™ ProGAV) utilized only from 2016. Key reasons for the preference of the MIETHKE™ ProGAV include the availability in a smaller size, the valve is MRI-resistant up to 3 Tesla and finally, the ability to ‘fine-tune’ the opening pressures. Hence, from 2016 onwards, we note a statistically significant shift in preferences for the CODMAN™ Certas and MIETHKE™ ProGAV PSVs, in comparison to the MEDTRONIC™ Strata. This was statistically significant (*p* < 0.001) on the Chi-square tests performed on the proportion of MEDTRONIC™ Strata PSV vs other PSV before, and after 2016. This change in practice likely explains the increase in the mean number of adjustments made per PSV after 2016. 

Following that, we observed that patients with PSVs that are shunt revisions tend to be adjusted more frequently than index shunts (2.2 mean adjustments per PSV, SD ± 1.9 versus 1.7 mean adjustments per PSV, SD ± 1.6; *p* = 0.488). In the cohort of patients with index shunts, the underlying etiology of hydrocephalus do not make a difference in terms of the mean shunt adjustments made per PSV. In addition, it is noted that patients whose hydrocephalus is tumor-related have their shunts adjusted more frequently compared to PSV patients secondary to other etiologies (2.3 mean adjustments per PSV; SD ± 1.6 versus 1.1 mean adjustments per PSV, SD ± 1.5; *p* = 0.107). Next, patients with PSV inserted during shunt revisions due to CSF over-drainage are adjusted more frequently than PSV implanted as shunt revisions for other causes (3.7 mean adjustments per PSV, SD ± 2.1 compared with 1.7 mean adjustments per PSV, SD ± 1.7; *p* = 0.231). Overall, analyses do not demonstrate statistical significance in the mean number of opening pressure adjustments made in our study cohort for the following comparisons: patients with index versus revision PSV shunts, between patients with different underlying etiologies for their index shunts; and those with CSF over-drainage secondary to a FPV and subsequently revised to a PSV. (Table 3).

## 4. Discussion

Optimal management of pediatric hydrocephalus remains contentious [26,27]. Published literature cites the failure rate of VPS can be up to 40% in the first year; and subsequent VPS revision rates lie between 40 and 60% [13,28]. For affected children with immature, developing brains, they bear the long-term risk of shunt-related surgeries throughout their lifetimes [29]. Traditionally, FPVs have been shown to be effective in the majority of patients [30]. Nonetheless, the chosen FPV for an individual patient may suboptimal, leading to CSF over-drainage due to siphoning effect or conversely, under-drainage—both potentially life-threating conditions [30]. Other longer-term risks include the development of slit-ventricle syndrome, loss of cerebral compliance [31,32,33], and occasionally, cranio-cephalic disproportion [34]. Inevitably, the selection of ideal working pressure of shunts remains challenging in children. The perfect shunt valve has yet to be designed, and no well-defined guidelines have been established for the selection of type of VPS [35].

### 4.1. Is There a Therapeutic Role for PSV in Pediatric Hydrocephalus?

The PSV is designed as part of efforts to avoid complications encountered in FPV [2]. Its key objective is to help regulate CSF flow and drainage better, in hopes of reducing the number of proximal shunt-related failures. These include slit ventricle syndrome, CSF under- and over-drainage [2]. Theoretically, the option of a PSV in situ provides the advantage of reducing the number of shunt revision procedures due to CSF drainage issues, as the shunt valve opening pressure can be adjusted non-invasively [6,36]. In contrast, repeat surgery (e.g., ligation of shunt tubing, or change of shunt valve) is typically necessary with the traditional use of FPV under such circumstances.

Although there are now more choices of PSV available by various manufacturers, there is no large-scale, randomized controlled trials performed to definitively compare efficacy between different types in children. Given the heterogeneity between them, neurosurgeons must rely on their own knowledge of the product, its theoretical benefits, and individual features to make a choice of the PSV used for their own patients. Specific to the pediatric population, preference is often given to the PSV that have the option of smaller sizes. This is because smaller-sized valves allow for the creation of smaller subcutaneous pockets that result in less overlying skin tension and lower rates of skin necrosis.

Extrapolating these factors in our institutional practice, our consensus is to consider the use of PSVs only in selected cases of pediatric hydrocephalus. As reflected in our study population, the main indications are uncertainty regarding the patient’s opening pressure, and or individuals who are expected to have changes in CSF dynamics over time. Examples of such include patients with slit ventricle syndrome, post-infectious hydrocephalus, those at risk of CSF over-drainage, or previously failed ETV. Following that, the choice of PSV is based on personal preference of each neurosurgeon. Deciding factors include the safety profile of the chosen PSV, physical size of PSV valves—in relation to cosmesis and wound healing in small children; the range of opening pressures and ability to ‘fine-tune’ pressure adjustments and level of MRI resistance.

In addition, our data compares favorably with reported data relating to shunt failure with 40% of children requiring intervention for shunt failure within the first 2 years after placement [37]. This is congruent with published results from previous studies whereby it has been demonstrated that the type of shunt valve used has no effect on shunt failure rates [13,38], with consensus guidelines not favoring any particular shunt valve type [18,39].

### 4.2. Quantifying Adjustments in PSV—Has Our Use Been Appropriate?

In our study population, the indication for adjustment of opening pressure was due to either a clinical indication (i.e., signs or symptoms suggestive of CSF under- or over-drainage), radiological findings of an interval change in the size of the ventricles (e.g., ventriculomegaly or slit-like ventricles). As part of our patient management, caregivers are routinely provided with detailed shunt education so they can remain vigilant on the signs and symptoms of drainage issues, especially in younger children. For the older, school-going patients who can report their own symptoms, their PSV settings may be adjusted on the basis of clinical findings and symptoms without the need for repeat neuroimaging. Our shunt adjustment data analyses suggest that the use of PSV at our institution has become more appropriate with time. More importantly, this subset of patients benefitted from the use of PSV, avoiding issues of repeated shunt revision surgery, in comparison to being implanted with FPV.

We note that the MEDTRONIC™ Strata valve is the most commonly used PSV in the early years of our practice. Over time, newer types of PSV that offer a wider range of opening pressure settings and higher MRI resistance become more popular. It has been shown that the PSV limits shunt dependent flow of CSF as the upgraded pressure activates the regular circulation of the CSF. Theoretically, this leads to cerebral development, and shunt removal will consequently be possible [40]. Takahashi et al. demonstrated that it is possible to remove the shunt systems in 50% or more of pediatric hydrocephalus cases in which PSV valves are used. This is achieved through careful control of the valve pressure [40]. Of interest, we observed that our threshold for PSV adjustments seemed to be lower after 2016. Upon reflection, the following changes are noted in recent years: firstly, closer surveillance of PSV patients; and there are more MRI brain scans ordered for individual patients, either by the primary clinician or co-managing subspecialists for various reasons. In this modern era of targeted treatment, we have encountered effective shrinkage of brain tumors within a short period of time [41,42]. Conversely, there are some instances whereby there is temporary brain tumor swelling during radiation therapy that cause symptoms of raised intracranial pressure. Under such circumstances, adjustments of the PSV alleviate the symptoms of these patients. Another example is the neonatal group whose head circumferences need to be monitored as their craniums are still actively growing. For these patients, we are cognizant their opening pressures are at risk of changes. Concurrently, the allowance of the ‘fine-tuning’ effect by the CODMAN™ Certas and MIETHKE™ ProGAV PSVs provides a gradual adjustment in suitable patients more confidently. This is especially so for those we hope that can achieve shunt independence eventually. As there are currently no established guidelines on the best approach to help children with PSV to wean off their shunts, we work closely with the patients and their caregivers to keep them on the qui vive.

### 4.3. Limitations of the PSV

Despite their versatility, there are complications unique to PSVs that are noteworthy. For example, the PSV contain intrinsic magnetic components that enable valve pressure to be changed using an external magnetic adjustment device [43]. These newer valves include locking systems that reduce the risk of unintentional changes to valve setting during MRI scans [44]. Among the 3 most commonly inserted PSV used by our unit, the CODMAN™ Certas and MIETHKE™ ProGAV shunts are deemed to be MRI-resistant by their manufacturers up to 3 Tesla [22,23]. Without this feature, the concern is that a patient’s PSV opening pressure setting may be adjusted inadvertently post-MRI exposure [44,45]. Even though all patients with PSV undergo MRI only in a 1.5 Tesla machine, it is our institutional protocol to still check their settings after every MRI is performed. Nonetheless, our preference has evolved to prefer the use of the newer valves with higher MRI resistance. At the time of PSV insertion surgery, each patient and their caregivers are given an instructional session on the product’s information. Reading material and personal shunt cards indicating the PSV settings and dates of shunt checks/ setting changes are also provided for them.

Following that, we are also aware that PSVs create artifact distortion that hamper the examination of brain structures [43,46,47]. An important disadvantage of such artefacts is that visible tumor may be obscured if the valve is placed too close by. In contrast, FPV have no risk of having the opening pressures being changed after exposure to magnetic fields, and do not require checking of the shunt settings after MRI scans. They also have minimal interference with MRI images [29]. Other problems associated with PSV reported in the literature include breakage secondary to minor head trauma [48], and malfunction due to in vivo wear and tear on the valve itself [5].

### 4.4. Study Limitations and Future Directions

At this point, the authors acknowledge that this is a study that is unique in the context of our local healthcare system. We are cognizant that equitable access to PSV in other parts of the world may differ; and that our approach may not apply in other countries where healthcare systems are different [49]. Following that, the close, long-term nature of clinical and, when necessary, radiological surveillance among our patients allow us to make pressure adjustments with greater confidence. Without corresponding similarities in treatment options, the “best outcomes” achievable in one geographic context may not necessarily apply in others [49,50]. Under such circumstances, we advocate an ‘individualized’ understanding of how patients may benefit from different shunt types will allow us to optimize patient outcomes; and to tailor a more personalized approach to managing pediatric hydrocephalus, rather than a ‘one-size-fits-all’ approach.

## 5. Conclusions

In summary, we describe our institutional experience of PSV use in pediatric hydrocephalus in a subset of patients whose opening pressures are uncertain and evolving. At the time of this writing, the use of PSV demonstrates potential for these patients to avoid the feared shunt-related complications of failure, over drainage and cerebral non-compliance. As the way forward, the authors advocate ongoing collaboration with international experts to seek better understanding of pediatric hydrocephalus, especially for selected patients whereby the underlying pathophysiology mechanisms are complex.

## Figures and Tables

**Table 1:** Opening pressures of selected fixed pressure shunts and programmable shunts [20,21,22,23,24,25]. (Of note, the standard setting for the Sophysa Polaris^®^ allows for opening pressures to be adjusted between 30 mmH2O and 200 mmH2O; additional options, however, are available: 10–140 mmH2O, 50–300 mmH2O, or 80–400 mmH2O.

	Medtronic PS Medical^®^ Strata^®^	Codman Certas Plus	MIETHKE proGAV^®^ 2.0	Codman Hakim	Sophysa Polaris
**Pressure range** **(mmH2O)**	15–220	25–400	0–200	30–200	30–200
**Number of settings**	5	8	20	18	5
**‘Virtual off’ function**	No	Yes	No	No	No
**MRI-safe**	Yes	Yes	Yes	Yes	Yes
**MRI-resistant**	No	Yes, up to 3 Tesla	Yes, up to 3 Tesla	No	No
**MRI artefacts**	Yes, worse at higher settings	Yes, worse at higher settings	Yes, worse at lower settings	Yes, based on in vitro findings	Yes, worse at higher settings
**Verification and adjustment of settings**	Portable, handheld adjuster tool	Portable, handheld adjuster tool	Portable, handheld adjuster tool	Requires X-ray verification	Portable, handheld adjuster tool
**Availability in Singapore**	Yes	Yes	Yes	Yes	No
**Availability in small sizes**	Yes	Yes, but not available in Singapore	Yes	Microvalve shunt available by manufacturer, but not available in Singapore	No

**Table 2:** Comparative mean number of adjustments per PSV before and after 2016.

	Before 2016	From 2016 Onwards	*p*-Value
Mean number of adjustments per PSV	0.8 SD ± 0.9	2.1 SD ± 1.7	*p* = 0.031

**Table 3:** Summary of comparison between subgroups of PSV patients.

**Comparison between Subgroups of PSV Patients**	**Mean Number of Adjustments per PSV**	***p*-Value**
Revision shunts versus index shunts	2.2; SD ± 1.9 1.7; SD ± 1.6	*p* = 0.488
Tumor-related versus other etiologies	2.3; SD ± 1.6	*p* = 0.107
	1.1; SD ± 1.5	
Previous CSF over-drainage shunts versus all other shunt revisions	3.7; SD ± 2.1	*p* = 0.231
	1.7; SD ± 1.7	

## Data Availability

Data is available on request.

## Supplementary Materials

The following are available online at https://www.mdpi.com/article/10.3390/brainsci11111548/s1, Figure S1: Failure-free survival compared between types of PSV, Figure S2: Failure-free survival comparison between index versus revision shunts, Figure S3: Histogram of frequency of PSV opening pressure adjustments.
