# Supplementary material for: Programmable Shunt Valves for Pediatric Hydrocephalus: 22-Year Experience from a Singapore Children’s Hospital
Source: Brain Sci. 2021 Nov 22;11(11):1548. doi: 10.3390/brainsci11111548 (PMC8615584; doi:10.3390/brainsci11111548)
Supplement: Supplementary file 1 [file brainsci-11-01548-s001.zip › brainsci-1420572-supplementary.pdf]

Supplementary Figures:

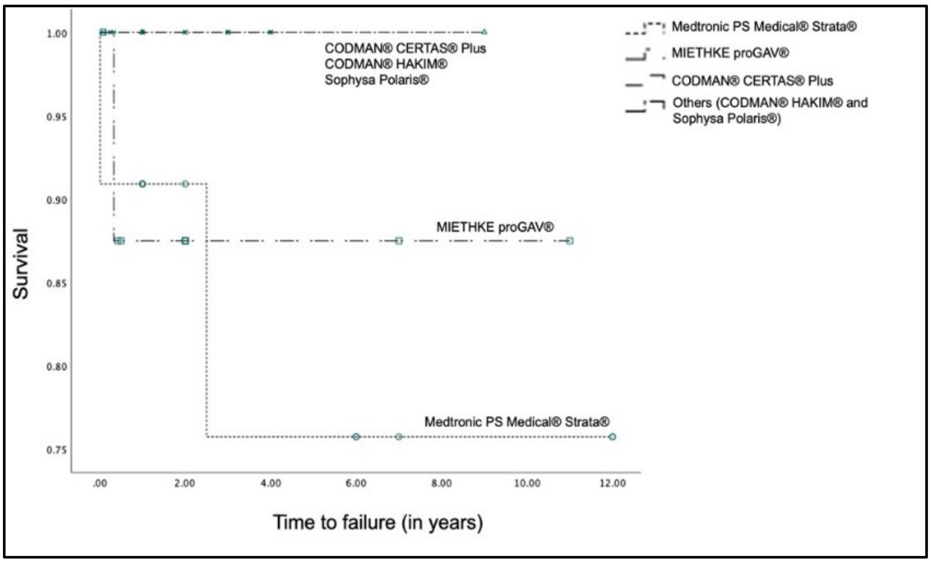

Figure S1. Failure-free survival compared between types of PSV.

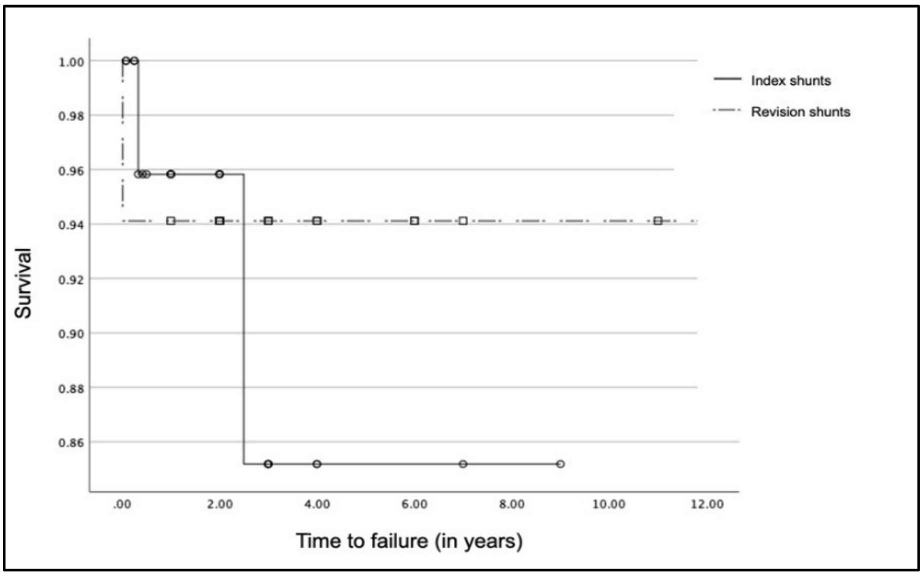

Figure S2. Failure-free survival comparison between index versus revision shunts.

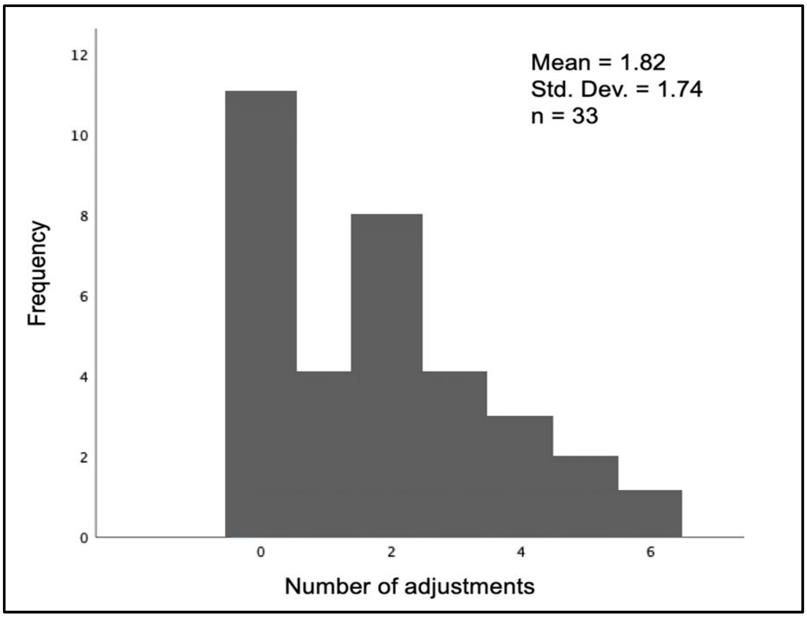

Figure S3. Histogram of frequency of PSV opening pressure adjustments.
